# Incidence of Underlying Abnormal Findings on Routine Magnetic Resonance Imaging for Bell Palsy

**DOI:** 10.1001/jamanetworkopen.2023.9158

**Published:** 2023-04-20

**Authors:** Thibault Savary, Maxime Fieux, Marion Douplat, Romain Tournegros, Sophie Daubie, Dylan Pavie, Luna Denoix, Jean-Baptiste Pialat, Stephane Tringali

**Affiliations:** 1 Service d’ORL, d’Otoneurochirurgie et de Chirurgie Cervico-Faciale, Hospices Civils de Lyon, Centre Hospitalier Lyon Sud, Pierre Bénite, France; 2 Université de Lyon, Université Lyon 1, Lyon, France; 3 Université Paris Est Créteil, Institut National de la Santé et de la Recherche Médicale (INSERM), Mondor Institute for Biomedical Research (IMRB), Créteil, France; 4 Centre National de la Recherche Scientiﬁque (CNRS) Equipe Mixte de Recherche 7000, Créteil, France; 5 Service des Urgences, Hospices Civils of Lyon, Centre Hospitalier Lyon Sud, Pierre Bénite, France; 6 Research on Healthcare Performance, Université Claude Bernard Lyon 1, INSERM U1290, Lyon, France.; 7 Unité Mixte de Recherche (UMR) Adés 7268, Aix-Marseille University, Etablissement Français du Sang–CNRS, Espace Éthique Méditerranéen, Marseille, France.; 8 Service d’Imagerie Médicale, Hospices Civils de Lyon, Centre Hospitalier Lyon Sud, Pierre Bénite, France; 9 Creatis CNRS UMR 5220, INSERM U1294, Université Lyon 1, Villeurbanne, France; 10 UMR 5305, Laboratoire de Biologie Tissulaire et d’Ingénierie Thérapeutique, Institut de Biologie et Chimie des Protéines, CNRS Université Claude Bernard Lyon 1, Lyon, France

## Abstract

**Importance:**

There is no consensus on the benefits of routine magnetic resonance imaging (MRI) of the facial nerve in patients with suspected idiopathic peripheral facial palsy (PFP) (ie, Bell palsy [BP]).

**Objectives:**

To estimate the proportion of adult patients in whom MRI led to correction of an initial clinical diagnosis of BP; to determine the proportion of patients with confirmed BP who had MRI evidence of facial nerve neuritis without secondary lesions; and to identify factors associated with secondary (nonidiopathic) PFP at initial presentation and 1 month later.

**Design, Setting, and Participants:**

This retrospective multicenter cohort study analyzed the clinical and radiological data of 120 patients initially diagnosed with suspected BP from January 1, 2018, to April 30, 2022, at the emergency department of 3 tertiary referral centers in France.

**Interventions:**

All patients screened for clinically suspected BP underwent an MRI of the entire facial nerve with a double-blind reading of all images.

**Main Outcomes and Measures:**

The proportion of patients in whom MRI led to a correction of the initial diagnosis of BP (any condition other than BP, including potentially life-threating conditions) and results of contrast enhancement of the facial nerve were described.

**Results:**

Among the 120 patients initially diagnosed with suspected BP, 64 (53.3%) were men, and the mean (SD) age was 51 (18) years. Magnetic resonance imaging of the facial nerve led to a correction of the diagnosis in 8 patients (6.7%); among them, potentially life-threatening conditions that required changes in treatment were identified in 3 (37.5%). The MRI confirmed the diagnosis of BP in 112 patients (93.3%), among whom 106 (94.6%) showed evidence of facial nerve neuritis on the affected side (hypersignal on gadolinium-enhanced T1-weighted images). This was the only objective sign confirming the idiopathic nature of PFP.

**Conclusions and Relevance:**

These preliminary results suggest the added value of the routine use of facial nerve MRI in suspected cases of BP. Multicentered international prospective studies should be organized to confirm these results.

## Key Points

**Question:**

What is the proportion of adult patients in whom magnetic resonance imaging (MRI) leads to correction of an initial clinical diagnosis of Bell palsy (BP)?

**Findings:**

In this retrospective cohort study of 120 patients with suspected clinical BP, routine MRI of the entire facial nerve in 8 patients (6.7%) resulted in identification of underlying causes, some of which could have been life-threatening.

**Meaning:**

These findings suggest that MRI in patients with suspected BP should be routine.

## Teaser

This cohort study estimates the proportion of adult patients in whom magnetic resonance imaging (MRI) led to correction of an initial clinical diagnosis of Bell palsy.

## Introduction

Peripheral facial palsy (PFP) is a common condition that affects 15 of 100 000 people per year.^1^ Idiopathic PFP, or Bell palsy (BP), is the most common PFP, accounting for 75% of cases, but PFP can also be caused by a variety of diseases.^2^ The main pathophysiological explanation for BP is postherpetic neuritis of the facial nerve.^3^ This cause is suggested by the absence of clinical or laboratory evidence of other possible causes.^4^ In most of these cases (85%), symptoms regress within a few weeks.^4^

In patients with BP, gadolinium-enhanced magnetic resonance imaging (MRI) of the facial nerve may reveal isolated neuritis of the facial nerve, seen as nonnodular contrast enhancement with no other associated abnormality, particularly in the cerebrum. This asymmetric enhancement (≥1 portion of the contralateral facial nerve may be slightly enhanced^5^) is the only objective marker of BP. It is found in approximately 60% to 90% of patients with BP and may weaken over time.^6,7^ Although it is not 100% sensitive for the diagnosis of BP, MRI focusing on the facial nerve can correct diagnostic errors in patients with PFP mimicking BP.^8^ While MRI in these cases has been recommended by the French Society of ENT (Ear Nose and Throat) and Head and Neck Surgeons since 2020 to avoid missing an underlying cause requiring different management,^4^ older US (2013)^9^ and Canadian (2014)^10^ guidelines argue against routine diagnostic imaging. Routine MRI of the facial nerve in patients with presumed BP is therefore controversial.

The main objective of this study was to estimate the proportion of adult patients in whom MRI led to correction of an initial clinical diagnosis of BP. The secondary objective was to determine the proportion of patients with confirmed BP who had MRI evidence of facial nerve neuritis without secondary lesions. A third objective was to identify factors associated with secondary (nonidiopathic) PFP at initial presentation and after 1 month.

## Methods

### Study Design and Ethics

This retrospective cohort study followed the Strengthening the Reporting of Observational Studies in Epidemiology (STROBE) reporting guideline. The study was conducted in accordance with the Declaration of Helsinki^11^ and was approved by the Institutional Review Board of Université de Lyon. Oral informed consent to participate was obtained from all patients. All patients received a letter of information regarding their data analysis as well as detailed contact information for declaring their refusal. All data were anonymized.

All medical records of adult patients who presented to the emergency departments of 3 tertiary hospitals in France with nontraumatic PFP between January 1, 2018, and April 30, 2022, were screened. Patients were included if they had an initial diagnosis of BP in the emergency department and subsequently underwent MRI (1.5T or 3T, with gadolinium enhancement). Following the establishment of the French guidelines in 2020, standardized clinical protocols are available in all emergency departments in the case of suspected BP. Emergency physicians should prescribe an MRI for the facial nerve and refer the patient to an ENT specialist within the first month after the onset of BP. The exclusion criteria for the present study were younger than 18 years, diagnosis other than BP, facial palsy sequelae still present before the current episode, diagnosis corrected by non-MRI examinations (laboratory tests, other examinations), lack of thin-section MRI of the facial nerve and/or lack of contrast enhancement, and refusal to participate in the study. The data collected regarding the patients’ medical history and follow-up as well as MRI features are available in eMethods in Supplement 1.

### Statistical Analysis

Patients were classified as having confirmed BP if their MRI findings confirmed the diagnosis of BP or as having nonidiopathic PFP if the initial diagnosis of BP was corrected based on MRI data. The 2 groups were compared to identify factors associated with nonidiopathic PFP at initial presentation and at the 1-month follow-up. The patients in the confirmed BP group were then subdivided according to whether their MRI findings were normal or pathological (hyperintensity in ≥1 portions of the facial nerve on the affected side). Finally, patients were grouped in terms of their time to MRI (<1 month or >1 month after initial presentation) to test the association with the presence of neuritis on MRI. Two-sided *P* ≤ .05 indicated statistical significance. All statistical analyses detailed in eMethods in Supplement 1 were performed with R software, version 4.1.2 (R Project for Statistical Computing).

## Results

### Population demographics

**Figure 1.**
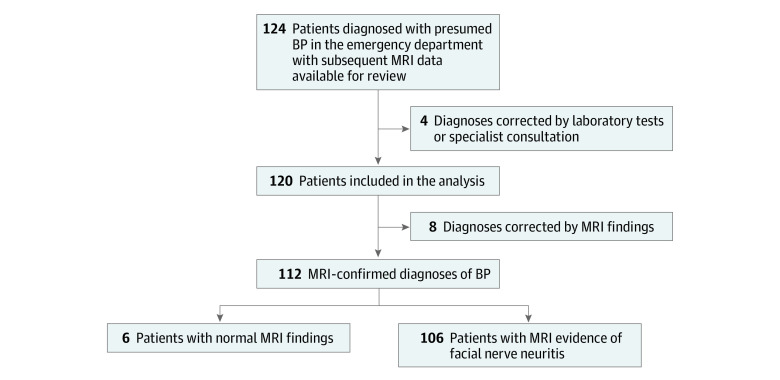
Study Flowchart BP indicates Bell palsy; MRI, magnetic resonance imaging.

This cohort study included 120 patients with a mean (SD) age at diagnosis of 51 (18) years; 56 patients (46.7%) were women and 64 (53.3%) were men. A total of 62 patients (51.7%) had right facial palsy; 19 (15.8%) had mild PFP (House-Brackmann [HB] grades II-III, indicating eye closure possible); and 100 (83.3%) had severe PFP (HB grades IV-VI, indicating no eye closure possible). Patient characteristics according to the final diagnosis are summarized in the Table. Initially, 573 patients presented to the 3 emergency departments with PFP between January 1, 2018, and April 30, 2022, among whom 504 (88.0%) were diagnosed with presumed BP during the initial consultation (emergency department visit). Among these patients, 14 (2.4%) underwent MRI of the brain, 35 (6.1%) underwent computed tomography of the brain, 63 (11.0%) first underwent computed tomography and then MRI of the brain, 149 (26.0%) underwent no cerebral imaging at all, and 312 (54.5%) underwent MRI of the facial nerve. Of these 312 patients, 124 (39.7%) had MRI data available for review. Four patients were excluded because the initial diagnosis was corrected by laboratory results or after referral to a specialist (2 cases of Lyme disease, 1 case of HIV seroconversion, and 1 case of autoimmune disease diagnosed by results of lumbar puncture). The study flowchart is shown in Figure 1.

**Table.** Patient Characteristics

Characteristic	Patient group^a^		*P* value
	Confirmed BP (n = 112)	Nonidiopathic PFP (n = 8)	
Sex			
Women	52 (46.4)	4 (50.0)	.99
Men	60 (53.6)	4 (50.0)	
Age, mean (SD), y	51 (18)	48 (16)	.77
Affected side			
Right	57 (50.9)	5 (62.5)	.72
Left	55 (49.1)	3 (37.5)	
Initial HB grade^b^			
II-III	15 (13.4)	4 (50.0)	.04
IV-VI	96 (85.7)	4 (50.0)	
History of PFP	6 (5.4)	2 (25.0)	.09
Type 2 diabetes	11 (9.8)	3 (37.5)	.05
Treatment			
Corticosteroids (1 mg/kg for 7-10 d)	107 (95.5)	8 (100)	>.99
Valaciclovir hydrochloride	73 (65.2)	3 (37.5)	.05
Lyme disease test	93 (83.0)	8 (100)	.35
HIV test	80 (71.4)	6 (75.0)	>.99
HSV or VZV test	51 (45.5)	4 (50.0)	>.99
Syphilis test	14 (12.5)	1 (12.5)	>.99
Rehabilitation	90 (80.4)	8 (100)	.54
ENT consultation	106 (94.6)	6 (75.0)	.09
Abnormal vocal/tonal audiogram results^c^	9 (11.0)	3 (33.3)	.09
Symptoms at 1 mo^d^			
Improvement	65 (73.0)	6 (75.0)	.26
No change	24 (27.0)	2 (25.0)	
Deterioration	0	0	
Time to MRI, mean (SD), d	26.6 (24.0)	28.1 (21.5)	.67
Follow-up, mean (SD), mo	18.9 (15.3)	6.8 (9.5)	.02

Abbreviations: BP, Bells palsy; ENT, ear, nose, and throat; HB, House-Brackmann; HSV, herpes simplex virus; MRI, magnetic resonance imaging; PFP, peripheral facial palsy; VZV, varicella zoster virus.

^a^ Unless otherwise indicated, data are expressed as No. (%) of patients. Percentages have been rounded and may not total 100.

^b^ Grades II to III indicate eye closure possible; grades IV to VI, eye closure not possible. The HB grade was missing for 1 patient in the confirmed BP group.

^c^ Assessed in 82 patients in the confirmed BP group and 3 in the nonidiopathic PFP group. Indicates decreased thresholds of at least 10 dB at 3 consecutive frequencies on the affected side compared with the healthy side.

^d^ Assessed in 89 patients in the confirmed BP group and 8 in the nonidiopathic PFP group. Improvement indicates lower HB grade at 1 month; deterioration, higher HB grade at 1 month.

### MRI Features Regarding Corrected Diagnosis

**Figure 2.**
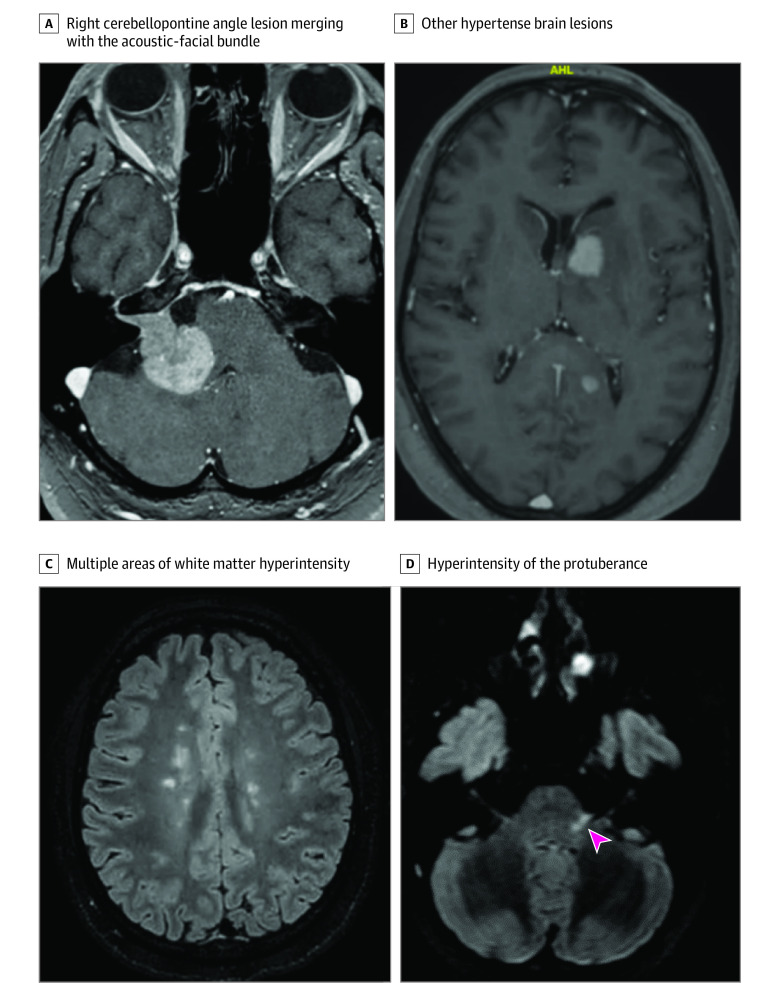
Magnetic Resonance Imaging of Patients With Peripheral Facial Palsy Due to Cerebral Lymphoma and Multiple Sclerosis A, Contrast-enhanced T1-weighted image in a patient in their 50s with right peripheral facial palsy (PFP) due to cerebral lymphoma showing right cerebellopontine angle lesion merging with the acoustic-facial bundle with enhancement after gadolinium injection. B, Contrast-enhanced T1-weighted image in the same patient showing other hypertense brain lesions. C, Multiple areas of white matter hyperintensity in a woman in her 20s with left PFP due to multiple sclerosis. D, Fluid-attenuated inversion recovery image in the same patient showing hyperintensity of the protuberance, seemingly in the root of the acoustic-facial bundle (arrowhead).

Eight patients (6.7%) with presumed BP had their diagnosis corrected as a result of MRI. Two patients had brain lymphoma with lesions in the nucleus and the first intracranial portion of the facial nerve on the affected side (Figure 2A and B). The first patient’s medical history was unremarkable. The second patient had type 2 diabetes and experienced a first episode of PFP 7 months earlier. Symptoms had fully regressed with corticosteroid treatment in 3 weeks. The MRI findings at the time were considered normal, but the examination was performed without contrast enhancement. The patient died 3 months after the onset of the second paralysis.

**Figure 3.**
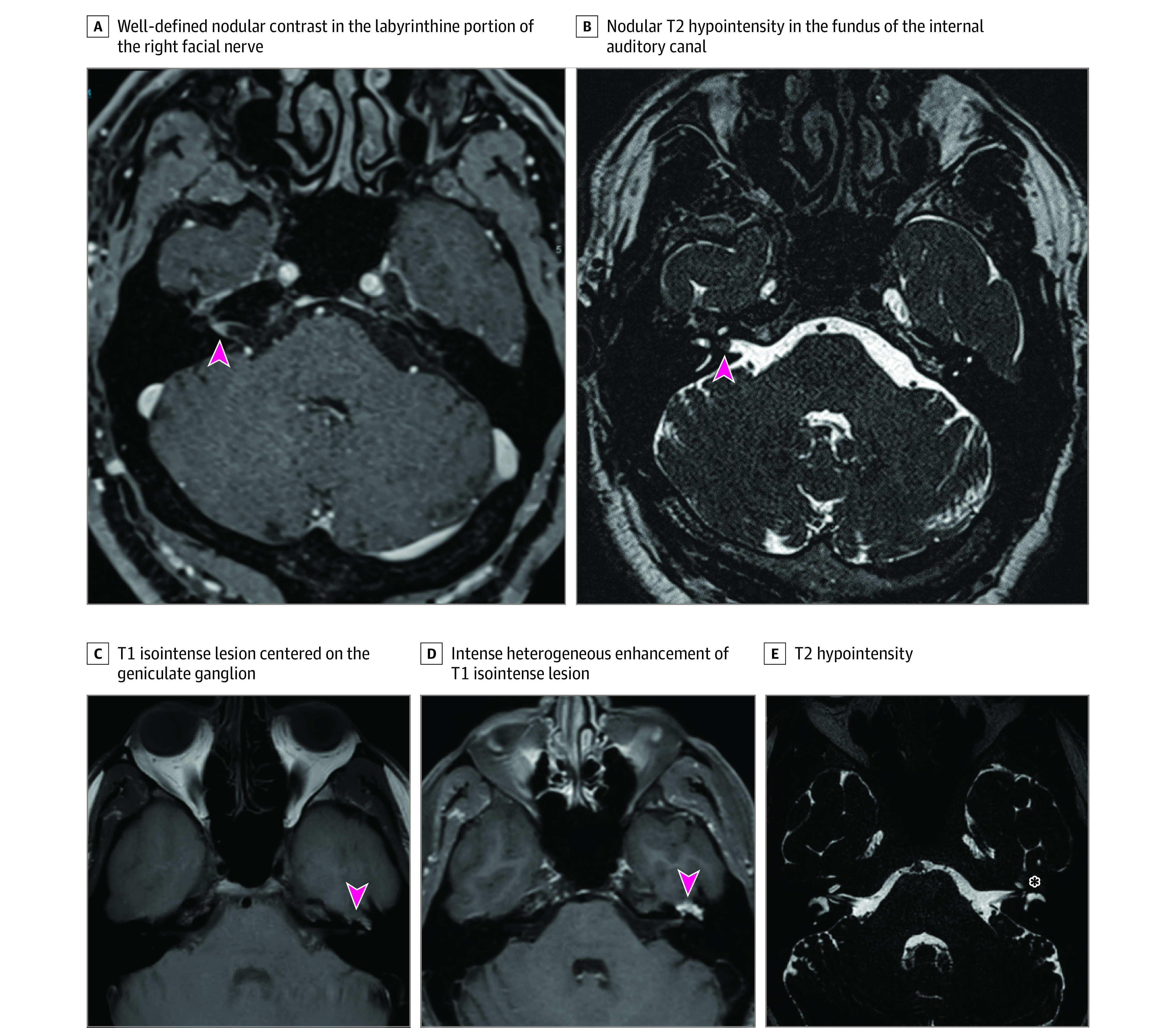
Magnetic Resonance Imaging of Patients With Peripheral Facial Palsy (PFP) Due to Facial Nerve Schwannoma and Facial Nerve Hemangioma A, Contrast-enhanced T1-weighted image in a patient in their 50s with right PFP due to facial nerve schwannoma showing well-defined nodular contrast in the labyrinthine portion of the right facial nerve (arrowhead). B, Nodular T2 hypointensity in the fundus of the internal auditory canal (arrowhead) in the same patient. C, T1 isointense lesion centered on the lateral part of the geniculate ganglion (arrowhead) in a patient in their 40s with left PFP due to facial nerve hemangioma. D, Intense heterogeneous enhancement after gadolinium injection (arrowhead) in the same patient. E, T2 hypointensity (asterisk) in the same patient.

Two patients had multiple white matter lesions, including 1 in the nucleus of the facial nerve on the affected side, leading to a diagnosis of multiple sclerosis (MS). No evidence of an alternative cause was identified, and subsequent imaging examinations confirmed the diagnosis (Figure 2C and D). Both patients showed total resolution of paralysis at 1 month with corticosteroid treatment. One patient had T1 hyperintensity in both facial nerves (slightly stronger on the affected side) and in the trigeminal nerve without associated lesions. A diagnosis of sarcoidosis or Wegener granulomatosis was made based on the involvement of multiple cranial nerves. The patient’s medical history was unremarkable except for congenital deafness and type 2 diabetes. Subsequent ophthalmologic findings (granulomatous posterior uveitis) and the observation of granulomas on results of a mediastinal lymph node biopsy confirmed the diagnosis of neurosarcoidosis. One patient had a homogenous hyperintense lesion centered on the facial nerve in the fundus of the internal auditory canal, indicative of facial nerve schwannoma on the affected side (Figure 3A and B). This patient had had a previous episode of presumed BP 4 years previously with a complete recovery. One patient with an unremarkable medical history had a homogenous hyperintense lesion centered on the eighth cranial nerve in the internal auditory canal, leading to a diagnosis of ipsilateral vestibular schwannoma. The final patient, also with an unremarkable medical history, had an irregular lesion centered on the geniculate ganglion and the second portion of the facial nerve, leading to a diagnosis of intraosseous hemangioma of the facial nerve (Figure 3C-E). Details regarding age, sex, medical history, and symptoms are available in eTable in Supplement 1.

### MRI Features Regarding Facial Nerve Enhancement

**Figure 4.**
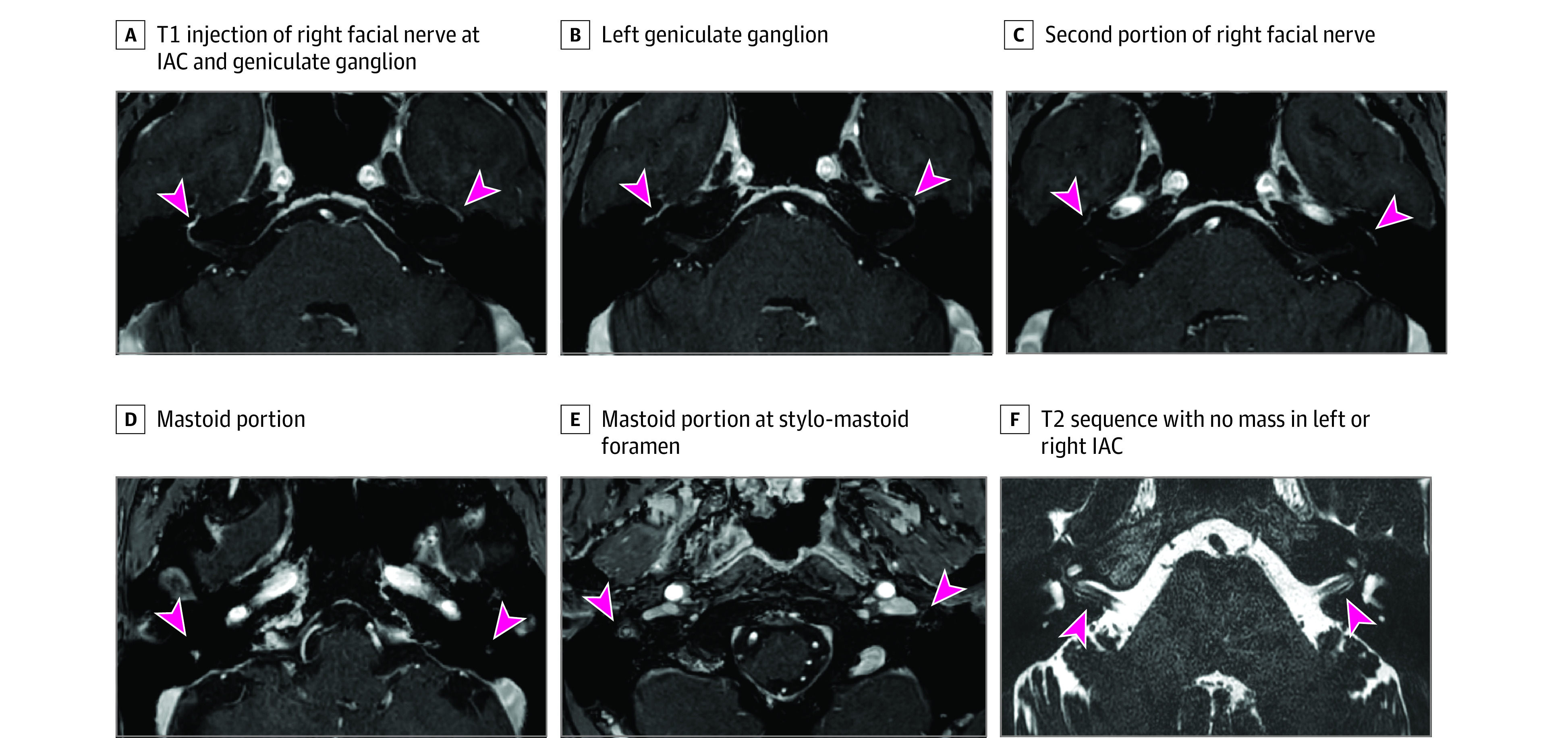
Magnetic Resonance Images of a Patient in Their 40s With Right Idiopathic Peripheral Facial Palsy Images were obtained 5 weeks after presentation to the emergency department, showing typical signs of right facial nerve neuritis. Left arrowheads indicate increased hyperintensity after gadolinium injection; right arrowheads indicate the left facial nerve for comparison. IAC indicates internal auditory canal.

Magnetic resonance imaging confirmed the diagnosis of BP in 112 of 120 patients (93.3%). Among these 112 patients, 106 (94.6%) had nonnodular ipsilateral facial nerve enhancement on MRI with no other associated abnormalities (Figure 4A-F). Hyperintensity in 1 or more segments of the facial nerve (in the first, second, or third segment of the nerve and/or the geniculate ganglion) was observed on the affected side in gadolinium-enhanced T1-weighted images. No lesions were observed in the nerve or in the parotid gland on submillimeter-resolution T2-weighted images, and no brain lesions were observed on fluid-attenuated inversion recover images. The MRI findings of the 6 remaining patients in the group with confirmed BP were considered normal. Follow-up was prolonged for these patients to confirm complete symptom resolution. For these 6 patients, BP was a diagnosis of exclusion in the absence of neuritis by MRI. Interobserver agreement between the 2 investigators (S.D. and D.P.) was excellent, with a Cohen κ coefficient of 0.92. No significant differences were identified between patients with normal MRI findings and those with neuritis in terms of age (mean [SD], 51 [13] vs 51 [18] years; *P* = .96), sex (1 of 6 [16.7%] vs 51 of 106 [48.1%] women; *P* = .21), HB grade (4 of 6 [66.7%] vs 92 of 106 [86.8%] with severe PFP [HB grades IV-VI]; *P* = .23), or initial treatment (5 of 6 [83.3%] vs 102 of 106 [96.2%] receiving corticosteroid treatment; *P* = .25). Magnetic resonance imaging was performed within 1 month for 3 of 6 patients (50.0%) without facial nerve enhancement and for 70 of 106 (66.0%) with evidence of neuritis. The remaining 36 of 106 patients (34.0%) underwent MRI between 1 and 4 months after initial presentation. The median time to MRI was 20.0 (IQR, 8.0-37.0) days in those without facial nerve enhancement vs 42.5 (IQR, 36.0-55.0) days in those with evidence of neuritis (*P* = .01). The median time to follow-up was 2.2 (IQR, 1.1-8.8) months in those without facial nerve enhancement vs 2.7 (IQR, 1.6-4.0) months in those with evidence of neuritis (*P* = .73).

### Factors Associated With Nonidiopathic PFP

Patients in the confirmed BP group were more likely to have incomplete eye closure than those in the nonidiopathic PFP group (proportions of patients with HB grades IV-VI, 96 of 112 [85.7%] and 4 of 8 [50.0%], respectively; *P* = .04). The proportion of patients with type 2 diabetes was significantly higher in the nonidiopathic PFP group (3 of 8 [37.5%] vs 11 of 112 [9.8%]; *P* = .05). Two of 8 patients (25.0%) in the nonidiopathic PFP group had a history of PFP compared with just 6 of 112 (5.4%) in the confirmed BP group (*P* = .09). The proportion of patients who received valacyclovir hydrochloride was significantly higher in the confirmed BP group (73 of 112 [65.2%] vs 3 of 8 [37.5%]; *P* = .05). Two of 8 patients with nonidiopathic PFP (25.0%; cases of hemangioma and facial schwannoma) showed no improvement in symptoms at 1 month, representing a similar proportion as in the confirmed BP group (24 of 89 [27.0%]; *P* = .26). There was no difference between the 2 groups of patients in terms of age (mean [SD], 48 [16] years in the nonidiopathic group and 51 [18] years in the confirmed BP group; *P* = .77), sex (4 of 8 [50.0%] women in the nonidiopathic group and 52 of 112 [46.4%] women in the confirmed BP group; *P* > .99), affected side (right side in 5 of 8 [62.5%] in the nonidiopathic group and 57 of 112 [50.9%] in the confirmed BP group; *P* = .72), subsequent ENT consultation (6 of 8 [75.0%] in the nonidiopathic group and 106 of 112 [94.6%] in the confirmed BP group; *P* = .09), audiogram assessment (abnormal audiogram assessment [ie, absence of stapedial reflex or hearing loss] in 1 of 3 [33.3%] in the nonidiopathic group and 9 of 82 [11.0%] in the confirmed BP group; *P* = .09), time to MRI (mean [SD] time between initial consultation and MRI, 28.1 [21.6] days in the nonidiopathic group and 26.6 [24.0] days in the confirmed BP group; *P* = .67), emergency department management (corticosteroid treatment in 8 of 8 [100%] in the nonidiopathic group and 107 of 112 [95.5%] in the confirmed BP group; *P* > .99), and use of rehabilitation (8 of 8 [100%] in the nonidiopathic group and 90 of 112 [80.4%] in the confirmed BP group; *P* = .54). Statistical data underlying these findings are available in the Table. The only type of pathological audiogram finding was sensorineural hearing loss.

## Discussion

In this group of patients with suspected BP, routine MRI of the entire facial nerve identified underlying causes in 8 of 120 cases (6.7%), some of which could have been life-threatening. Asymmetric enhancement of 1 or more segments of the facial nerve (labyrinthine, tympanic, mastoid, and/or geniculate ganglion) was quasi-pathognomonic of BP, being identifiable in 106 of 112 patients with confirmed BP (94.6%). Apart from the severity of impairment (96 of 112 [85.7%] of patients with HB grades IV to VI in the confirmed BP group vs 4 of 8 [50.0%] of those in the nonidiopathic PFP group; *P* = .04) and type 2 diabetes (11 of 112 [9.8%] in the confirmed BP group vs 3 of 8 [37.5%] in the nonidiopathic PFP group; *P* = .05), none of the studied factors was associated with an underlying cause. These results therefore support the 2020 French guidelines^4^ regarding the added value of MRI within a month of symptom onset^1^ in all patients with suspected BP, in addition to the standard clinical and laboratory workup to rule out differential diagnoses.

In contrast, the 2014 US guidelines^10^ recommend that MRI should only be used in patients with PFP who do not respond to treatment. However, a 2013 observational registry study^12^ found that performing MRI was significantly associated with revised diagnosis in suspected cases of BP. Fahimi et al^12^ did not investigate whether MRI alone led to a correction of the diagnosis, but their results are consistent with ours. Indeed, 6.7% of patients were found to have been initially misdiagnosed with BP and had a change in treatment based on MRI findings. Based on immediate MRI and lumbar puncture findings, Zimmermann et al^13^ found similarly that 30 of 509 cases of PFP (5.9%) were secondary to a tumor or autoimmune disease. However, since imaging was performed immediately without waiting for other initial workup results, the proportion of patients who would have been misdiagnosed with BP in the absence of MRI would perhaps have been lower. Autoimmune- and tumor-related PFP can both be misdiagnosed as BP.^14^ Regarding autoimmune diseases, 2 existing studies^15,16^ also support the routine use of MRI in patients with suspected BP. Di Stadio et al^15^ observed that 13 of their 18 patients with MS (72.2%) had a history of PFP with no other associated clinical sign, with PFP being the first symptom of MS in 3 of the 13 cases (23.1%). Therefore, PFP can be caused by central lesions and be misdiagnosed as BP in early stages of the disease.^15^ Similarly, Nwebube et al^16^ found that 23 of 218 patients with neurosarcoidosis (10.6%) in their cohort had PFP, with PFP being the first and only symptom of the disease in 12 of 23 cases (52.2%). Regarding tumors as the underlying cause, 50% of patients with facial nerve schwannoma have PFP,^17^ and Li and Dai^18^ found that 11 of their 32 patients with facial nerve schwannoma (34.4%) were initially diagnosed with BP before MRI. Meanwhile, Matthies and Samii^19^ found that 17% of patients with vestibular schwannoma had PFP. Peripheral facial palsy was also reported in nearly all cases (113 of 120 [94.2%]) of facial nerve hemangioma reviewed by Oldenburg et al^20^ and as an isolated symptom in more than 50% of cases. As reviewed recently by Bacrorn et al,^8^ numerous other diseases can mimic BP, a substantial proportion of which (4 of 25 [16.0%]) involve the parotid gland. Thin sections of the parotid glands are, therefore, essential in imaging protocols to avoid missing an underlying parotid disease.^21^ Finally, isolated PFP can also be caused by ischemic or hemorrhagic lesions in the pons involving the nucleus of the facial nerve,^22^ but these lesions rarely manifest as PFP alone.^23^ One of the benefits of MRI in cases of presumed BP is the elimination of causative diseases with clinical presentations that mimic those of BP.^24,25,26,27,28,29^

Another reason for performing MRI in presumed BP is to confirm the diagnosis, which is particularly important in view of the major effect of PFP on quality of life.^24^ While until recently, BP was a diagnosis of exclusion, based on the absence of other clinical or laboratory abnormalities,^4^ the advent of cranial nerve MRI^25^ now allows BP to be diagnosed positively. Almost all of the patients in our study (106 of 112 [94.6%]) with confirmed BP had asymmetric MRI enhancement of at least 1 segment of the facial nerve in agreement with the proportions reported by Kinoshita et al (114 of 125 [91.2%]),^7^ Seok et al (51 of 57 [89.5%]),^26^ and Kohsyu et al (22 of 22 [100%]).^27^ Although some authors found that the level of contrast enhancement decreased with increasing time to MRI,^6,27^ no such association was observed herein (3 of 6 [50.0%] of contrast enhancement at <1 month and >1 month among patients without neuritis found on MRI). Moderate facial nerve neuritis is sometimes difficult to observe on MRI, with potential disagreement between different investigators.^28^ In our study, however, interobserver agreement was excellent (κ = 0.92). The proportion of cases of neuritis observed on MRI in the same series of patients has also been found to depend on the type of sequence used.^29,30,31^

Among the variables tested herein as potential associated factors, initial severity of the impairment and type 2 diabetes status were significantly associated with MRI evidence of causative disease. Patients in the nonidiopathic PFP group were more likely to have less severe symptoms at presentation (HB grade II or III). A low HB grade in BP is usually considered reassuring and a prognostic factor associated with good recovery.^32^ However, our results indicate that MRI should still be performed in these cases. The prevalence of type 2 diabetes in the confirmed BP group (11 of 112 [9.8%]) is similar to the value reported by Jeong et al (204 of 2708 [7.5%]).^33^ However, the fact that type 2 diabetes was more prevalent in the nonidiopathic PFP group indicates that this well-established risk factor for BP is not conclusive evidence of an idiopathic origin. There was no significant difference in symptom improvement at 1 month between the 2 groups. This may be because several causes of nonidiopathic PFP are responsive to corticosteroid therapy (ie, MS, neurosarcoidosis, cerebral lymphoma), such that facial function may improve at 1 month.^15,16,34^ Corticosteroids are recommended for patients in whom there is any suspicion of BP, and they improve the prospect for recovery.^35^ Rapid improvement of PFP symptoms, while typically considered reassuring, is not synonymous with BP or a reason not to perform MRI.^36^ The percentage of patients with a history of PFP (5.4%) in the confirmed BP group was similar to the value reported by Peitersen et al^2^ (6.8% [No. of patients not reported]). Although this proportion was higher in the nonidiopathic PFP group (2 of 8 [25.0%]), this difference was not significant (*P* = .09). Regarding audiometry findings, the proportion of patients in the confirmed BP group with abnormal results (9 of 82 [11.0%]) is in agreement with previously reported values of 3 of 51 (5.9%) and 9 of 24 (37.5%) patients having ipsilateral abnormalities.^37,38^

There is currently no international consensus on this subject,^4,9,10^ but our findings on the prevalence of secondary PFP among patients with presumed BP and on the sometimes life-threatening nature of the underlying cause support the French stance of recommending MRI in all such cases. This work also highlights that symptom improvement and type 2 diabetes are not confirmatory of BP. Given the high cost of complementary examinations such as MRI, it would be interesting to conduct a cost-benefit analysis. Indeed, from a French perspective, the health care system may support the implementation of such strategy, but this may not be the case in other countries where insurance policies may differ. Therefore, the cost-benefit ratio of this strategy must be evaluated by future studies.

### Limitations

The limitations of this study include the low proportion of patients for whom MRI data were available and the variability of MRI protocols and devices. Indeed, this was a retrospective study with much missing data, specifically regarding MRI availability, which could represent a bias. However, the proportion of patients with nonidiopathic BP is very similar to the proportions reported in the literature,^12,13^ and the excellent interobserver agreement between the 2 senior radiologists’ interpretations suggests that the results of this work are reproducible and reliable.

## Conclusions

To our knowledge, this cohort study is the largest to date suggesting the added value of the routine use of MRI in cases of suspected BP. In this group of patients, asymmetric contrast enhancement on MRI was quasi-pathognomonic of BP, while clinical signs, symptom evolution, and type 2 diabetes were not unambiguously associated with BP. Multicentered international prospective studies should be organized to confirm these results.

## Notes

Supplement 1. **eMethods.** Study Population and Analysis **eTable.** Characteristics of Patients With Nonidiopathic PFP

Supplement 2.
Data Sharing Statement
