# Supplementary material for: Incidence of Underlying Abnormal Findings on Routine Magnetic Resonance Imaging for Bell Palsy
Source: JAMA Netw Open. 2023 Apr 20;6(4):e239158. doi: 10.1001/jamanetworkopen.2023.9158 (PMC10119737; doi:10.1001/jamanetworkopen.2023.9158)
Supplement: Supplement 1. — eMethods. Study Population and Analysis eTable. Characteristics of Patients With Nonidiopathic PFP [file jamanetwopen-e239158-s001.pdf]

Supplementary Online Content

Savary T, Fieux M, Douplat M, et al. Incidence of underlying abnormal findings on routine magnetic resonance imaging for Bell palsy. *JAMA Netw Open*. 2023;6(4):e239158. doi:10.1001/jamanetworkopen.2023.9158

eMethods. Study Population and Analysis

eTable. Characteristics of Patients With Nonidiopathic PFP

This supplementary material has been provided by the authors to give readers additional information about their work.

© 2023 Savary T et al. *JAMA Network Open*.

eMethods. Study Population and Analysis

Population

The data collected were the patients' medical history (history of PFP or diabetes), whether corticosteroids and/or antivirals were administered, and serological test results if prescribed (Lyme disease, herpes simplex virus, varicella zoster virus, human immunodeficiency virus (HIV), and syphilis). The degree of nerve damage was graded during the initial consultation (emergency department visit) using the House and Brackmann (HB) classification as grade 2-3 if the patient could close the eye on the affected side or grade 4-5-6 if they could not. Symptom improvement was defined as positive if the HB grade was lower at the one-month follow-up (ENT visit) than at initial presentation (emergency department visit). The results of subsequent ENT consultations and examinations were recorded: tonal and vocal audiometry and tympanometry with a stapedial reflex test. Audiogram results were considered pathological (sensorineural or conductive hearing loss) if they showed decreased thresholds of at least 10 dB at three consecutive frequencies on the affected side compared with the healthy side.

MRI analysis

The facial nerve was explored on millimeter-resolution gadolinium-enhanced T1-weighted images centered on the internal auditory canal (IAC) (volumetric interpolated breath-hold examination (VIBE), Philips T1-weighted high-resolution isotopic volume examination (THRIVE), or General Electric CUBE sequences) and submillimeter-resolution T2-weighted images. The MRI also included gadolinium-enhanced FLAIR sequence on the whole brain and parotid slices in T1-weighted images or T2-weighted images extending at least to the branch point of the facial nerve.

Patients without MR images of the entire parotid gland but for whom the entire course of the facial nerve trunk could be explored up to the branch point were retained. Patients who underwent vascular MRI only (stroke-type protocol with diffusion sequences, MR angiography of the circle of Willis, no thin slices of the facial nerve) were excluded. MRI parameters (matrix size, slice thickness, spin echo time) varied between patients because the data were acquired at 1.5 or 3 Tesla both in the study centers and externally. The MR images were saved on a single picture archiving and communication system (PACS) and were all analyzed independently by two senior radiologists using the same software (double-blind reads for all). The two experts reading the MR images were blinded to participant data and to each other's analysis. In the case of a dispute, the blind was lifted, and images were re-read by the two radiologists with the ENT specialist. Facial nerve neuritis was defined as nonnodular contrast enhancement of the affected facial nerve, with no other associated cerebral or parotid abnormalities. The time to MRI after initial presentation was also recorded.

Statistical analysis

Categorical variables are expressed as frequencies and percentages and were compared between groups using chi square tests (or Fischer's exact test when the former was not applicable). Continuous variables are summarized as the means and standard deviations (except when data were not normaly distributed, median and quartiles) and were compared between groups using the Kruskal-Wallis test because the sample size in one of the two groups was insufficient for Student's t test. The threshold for statistical significance was 0.05. Interobserver agreement was assessed using Cohen's Kappa coefficient for a binary classification (normal or neuritis) without weighting either of the two experts. All statistical analyses were performed with R software (v. 4.1.2, R Foundation for Statistical Computing, Vienna, Austria, www.r-project.org).

© 2023 Savary T et al. *JAMA Network Open*.

eTable. Characteristics of Patients With Nonidiopathic PFP

**Total (n=8)**	**Brain lymphoma (n=2)**	**Multiple sclerosis (n=2)**	**Sarcoidosis (n=1)**	**Benign tumor (n=3)**
**General characteristics**	% (No.)	% (No.)	% (No.)	% (No.)
Female sex	50 (1/2)	100 (2)	100 (1)	
Age (years, mean (SD))	54 (±5)	23 (±6)	56	58 (±10)
**Medical history**				
History of PFP	50 (1)	0 (0)	0 (0)	33.3 (1)
Diabetes	50 (1)	0 (0)	100 (1)	0 (0)
**Subsequent symptoms**				
Other than PFP	None	None	Uveitis	None
**One-month evolution**				
Resolution	100 (2)	100 (2)	100 (1)	33.3 (1) vestibular schwannoma
No change	0 (0)	0 (0)	0 (0)	66.7 (2) other tumors*
Deterioration	0 (0)	0 (0)	0 (0)	0 (0)
**Final diagnosis**				
Histology				Vestibular schwannoma (n=1)<br>*Facial nerve schwannoma (n=1)<br>*Hemangioma of the facial nerve (n=1)

© 2023 Savary T et al. *JAMA Network Open*.
