# Supplementary material for: Incidence of Underlying Abnormal Findings on Routine Magnetic Resonance Imaging for Bell Palsy
Source: JAMA Netw Open. 2023 Apr 20;6(4):e239158. doi: 10.1001/jamanetworkopen.2023.9158 (PMC10119737; doi:10.1001/jamanetworkopen.2023.9158)
Supplement: Supplement 2. — Data Sharing Statement [file jamanetwopen-e239158-s002.pdf]

Data Sharing Statement

Savary. Incidence of Underlying Abnormal Findings on Routine Magnetic Resonance Imaging for Bell Palsy. *JAMA Netw Open*. Published April 20, 2023.
doi:10.1001/jamanetworkopen.2023.9158

Data
Data available: No

Additional Information
Explanation for why data not available: Anonymous data could be shared on reasonable request
